# Salivary IL-8, IL-6 and TNF-α as Potential Diagnostic Biomarkers for Oral Cancer

**DOI:** 10.3390/diagnostics7020021

**Published:** 2017-04-09

**Authors:** Haafsa Arshad Sahibzada, Zohaib Khurshid, Rabia Sannam Khan, Mustafa Naseem, Khalid Mahmood Siddique, Maria Mali, Muhammad Sohail Zafar

**Affiliations:** 1 Department of Oral Medicine, Islamabad Dental Hospital (IDH), Islamabad 44000, Pakistan; Haafsa.OralMed16@iideas.edu.pk; 2 Department of Prosthodontics and Implantology, College of Dentistry, King Faisal University, Al-Ahsa 31982, Saudi Arabia; 3 Department of Oral Pathology, College of Dentistry, Baqai University, Super Highway, P.O. Box 2407, Karachi 74600, Pakistan; rabia.sannam@baqai.edu.pk; 4 Department of Preventive Dental Sciences, College of Dentistry, Dar-Al-Uloom University, Riyadh 13314, Saudi Arabia; m.naseem@dau.edu.sa; 5 Department of Oral Maxillofacial Surgery and Oral Medicine, Islamabad Dental Hospital (IDH), Islamabad 44000, Pakistan; drkhalidms@gmail.com; 6 Department of Periodontology, Fatima Jinnah Dental College, Karachi 74000, Pakistan; drmariahmali@gmail.com; 7 Department of Dental Materials, College of Dentistry, Al-Taibah University, Madinah Munawwarah 41331, Saudi Arabia; MZAFAR@taibahu.edu.sa; 8 Department of Dental Materials, Islamic International Dental College, Riphah International University, Islamabad 44000, Pakistan

**Keywords:** biopsy, saliva, biomarker, interleukin-8, interleukin-6, tumor necrosis factor-α (TNF-α), cancer and salivaomics

## Abstract

Saliva has been useful as a liquid biopsy for the diagnosis of various oral or systemic diseases, and oral squamous cell carcinoma (OSCC) is no exception. While its early detection and prevention is important, salivary cytokines expression, specifically of Interleukin-8 (IL-8), Interleukin-6 (IL-6) and Tumor necrosis factor (TNF-α), does contribute to the pathogenesis of cancer and these cytokines serve as potential biomarkers. Their excessive production plays a role in cancer progression and establishment of angiogenesis. However, other inflammatory or immunological conditions may affect the levels of cytokines in saliva. This article reviews the expression of levels of specific cytokines i.e., IL-8, IL-6 and TNF-α, their signaling pathways in the development of oral cancer, and how they are essential for the diagnosis of OSCC and updates related to it. Apart from serum, the saliva-based test can be a cost-effective tool in the follow-up and diagnosis of OSCC. Moreover, large-scale investigations are still needed for the validation of salivary cytokines.

## 1. Introduction

Oral cancer was previously rated as the sixth most common cancer worldwide, and has now acquired the fifth position [1]. Living in 21st century, with various scientific advancements being explored and discovered each day, the outcome of oral cancer worldwide has yet a prognosis which has deteriorated rather than improved. It is an established public health care problem with high mortality and morbidity rate [2]. Biopsy was known as the ultimate ′gold standard’ for the diagnosis of oral cancer [3]. Different techniques are being implemented to predict the development of oral cancer. These include vital staining, brush biopsy, auto-fluorescence spectroscopy, chemiluminescent illumination, narrow band imaging and confocal microscopy [3]. These methods not only prove to be invasive, but high-quality devices are needed to complete the biopsies, which turns out to be costly and cumbersome. Moreover, they only prove positive if dysplastic changes have occurred. To predict the nature of this lethal disease, more sensitive, non-invasive, cost-effective, and patient-friendly methods that are easily collectable and transferable for analysis tests are required [4].

Body fluids including blood, cerebrospinal fluid, urine, sweat, tears, saliva, peritoneal fluid, vomit, breast milk, semen, vaginal fluids, and drainage fluids have unique structural composition for specific conditions and disease [5]. Recently, literature published on the diagnostic capabilities of gingival crevicular fluid (GCF) has been reported by our group and we highlighted the importance of GCF as a diagnostic fluid for oral diseases detection, specially gingivitis and periodontitis [6]. Amongst these fluids, saliva is now being assessed as a predictive, diagnostic and prognostic tool for carcinomatous, inflammatory, and genetic diseases, among others [7]. The diagnostic potential of saliva is not limited to the diseases of the oral cavity, but covers systemic conditions as well [8]. Human saliva contains proteins, peptides, hormones and enzymes, each of which can easily be used to assess different diseases [9,10,11]. Saliva acts as a reservoir of steroids, amines, peptides, melatonin, insulin, leptin, ghrelin, secretory IgA enzymes, other enzymes, and drugs [12]. Approximately 1400–2000 proteins have been identified in saliva [13,14]. The number of proteins in saliva shows its diversity, and each of these proteins can be used as a simple tool to assess toxicity, infectious, immunological and hormonal levels [15]. Human saliva represents whole body images and is also known as the “mirror of the body”. Through the analysis of its resting flow rate (RFR) and pH on tobacco user subjects, it has been concluded that pH is affected by tobacco, but not RFR [16,17] A biomarker is a measurable nano-protein which is used to predict a biological state. With the extensive research on oral cancer, emphasis has been laid on predictive biomarkers found in saliva. Interestingly, positive values have been found. Glutathione, tissue polypeptide antigen (TPA), MRP14, interleukins, CD44, CD59, mac-2 binding protein (M2BP), immunoglobulin gamma (IgG), p53 antibodies, profilin, S100 calcium binding protein, endothelin-1, albumin, telomerase, cyfra 21-1, cancer antigen 125 (CA-125), transferrin, fibrin, salivary zinc finger, cofilin-1, protein 510 peptide, amylase, keratin 36, cystatin A, truncated cystatin SA-I, myosin, actin, ,S100A7, keratin-19 and catalase, signal transducer and activator of transcription-3 (STAT3), serpin B3 (SCCA1), α-1-antitrypsin (AAT), haptoglobin (HAP), thioredoxin, KNG1, ANA2, and Heat Shock Protein Family A 5 (HSPA5), tobacco-specific nitrosamines (TSNAs), *N*′-nitrosonornicotine (NNN), HAP, secretory leukocyte peptidase inhibitor, keratin 36, and cystatin A are some of the proteins identified, amongst 3000 others, in the detection, prognosis and prediction of oral cancer with variable sensitivity and specificity [2,12,18,19]. Unfortunately, to date, no single marker has yet been agreed upon due to lack of research and consensus amongst researchers. To find a biomarker that could predict or diagnose the disease as early as possible is the aim of researchers at the moment. Salivary biomarkers are either proteomic or genomic macromolecules in saliva. However, cytokines have always been regarded as superior amongst the wide range of biomarkers tested. Cytokines are a small group of secreted proteins which are labelled as non-structural proteins involved not only in inflammation, where they induce the growth, proliferation, and differentiation of normal cells, but also in tumorigenesis [20]. Amongst these, IL-6, IL-8, and TNF-α have been investigated in various conditions repeatedly. Of these three, IL-8 was regarded as the prototype of this group.

## 2. Salivary IL-8, IL-6 and TNF-α

**Figure 1:**
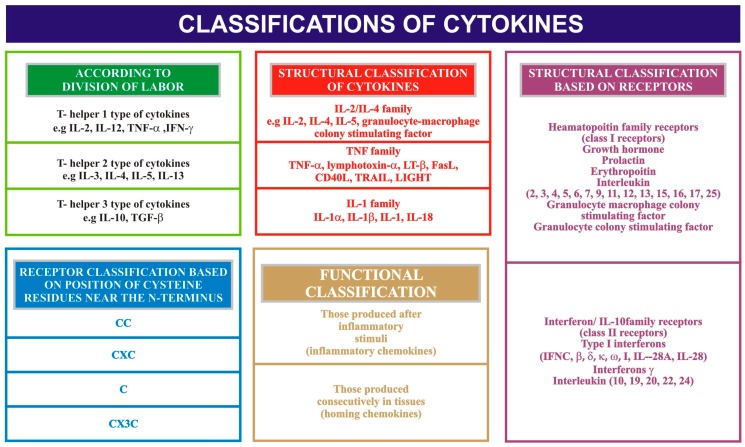
Classification of cytokines. IL = Interleukin; TNF = Tumor necrosis factor; IFN = Interferon; TGF = Transforming growth factor; TRAIL = TNF-related apoptosis inducing ligand; LIGHT = homologous to Lymphotoxin, exhibits Inducible expression and completes with HSV Glycoprotien D for binding to Herpesvirus entry mediator, a receptor expressed on T-lymphocytes; CC, CXC = conserved cysteine residues and chemokines; IFNC = Interferon-C.

Immune and non-immune cells of the human body produce low molecular weight glycoproteins, which are important regulators of the immune processes. Of glycoproteins, the cytokine group has gained attention primarily because of its critical role in regulating, signaling, maintaining and inducing most of the cellular interactions; they are hence regarded as molecular messengers [21]. The function of cytokines includes inflammation, apoptosis, host resistance, hematopoietic, and immunological responses, amongst many others. Characteristic features of cytokines include pleiotrophism i.e., they have the ability to interact not with one another but with other cellular targets, and cytokine crossover, i.e., a single cellular target can respond to multiple cytokines. They are chemotactic in nature, i.e., they attract particular cells via a concentration gradient. The doses of cytokines are generally maintained within a specified range and time. If not properly maintained, they can lead to induction of tissue damage. Cytokines can be classified according to the division of labour, on a structural basis and based on receptors (see Figure 1). Systemic conditions including obesity, psoriasis, anaphylactic shock, Steven-Johnson syndrome and other acute inflammatory conditions are known to increase the levels of cytokines several times over. Oral conditions, specifically oral aphthous ulcers, oral dysplastic lesions, oral and pharyngeal carcinomas and periodontitis, are known to contribute to elevated levels of cytokines as well.

IL-8 is regarded as the prototype of the chemokine family. It is pro-inflammatory in nature and is released by the neutrophils and macrophages in response to various stimuli, including chemical environment, steroids, inflammatory signals and environmental stresses. These stimuli activate the nuclear factor-kappa-B (NF-κB) pathway and this, in turn, activates the expression of IL-8 production. The IL-8 produced acts on two structurally similar but antigenically different receptors, namely CRCX-1 and CRCX-2. These receptors are located on tumor-associated macrophages, neutrophils and cancer cells. The presence of the receptors on cancer cells strongly suggests that the levels of IL-8 are an important chemokine for cancer cells microenvironment [22]. The pathogenicity in cancer cells is derived from neutrophil recruitment, angiogenic potential, proliferation, survival, migration of vascular endothelial cells, protection from apoptosis and, ultimately, metastasis [23]. The role in cancer can also be postulated from the fact that treatment of cancerous lesions with chemotherapeutic agents like 5-fluorouracil, among many others, reduces the expression of IL-8 [18,24]. The stimulating agents, if removed or decreased, also alter the IL-8 expression and levels, which further provide confirmation. Moreover, Selvam et al. suggested salivary 1L-6 as a diagnostic marker for leukoplakia and OSCC by determining its concentration by enzyme-linked immunosorbent assay (ELISA), the results of which proved its high concentration and production by tumor cells [25]. Juretic et al. suggested and confirmed through ELISA the high levels of IL-6 and TNF-α, proving their diagnostic and prognostic significance in potentially premalignant lesions and conditions and in OSCC [26]. Nelson et al. suggested the elevation of IL-6, IL-8, IL-1 and TNF-α in oral neoplastic lesions and OSCC because of their characteristic feature involvement in pro-angiogenesis and pro-inflammation which, in turn, has diagnostic value [27].

**Figure 2:**
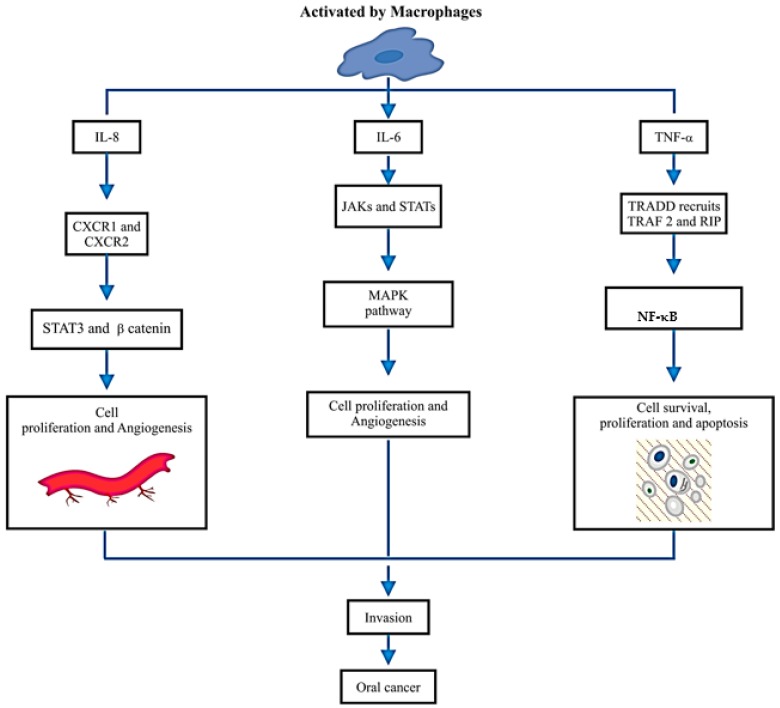
A schematic diagram illustrates the activation of IL-8, IL-6 and TNF-α through activated macrophages and the range of their signalling pathways in tumor microenvironments.

IL-6, apart from being a pro-inflammatory cytokine, also regulates regenerative, metabolic and neural processes. It follows the ras/raf/Mitogen-activated protein (MAP) kinase (MAPK) pathway [28]. TNF-α involves caspase cascades, transcription factors, nuclear factor kappa B (NF-κB) and activating protein 1 (AP-1), which are involved in inflammation, signal regulation, cell growth and death [29]. IL-8, IL-6 and TNF-α proved to be overexpressed in OSCC, as investigated by the University of California, Los Angeles (UCLA) Collaborative Oral Fluid Diagnostic Research Centre, the pioneer center for the studies conducted in this field [30]. Keeping in view the above, various researchers have been adamant on concluding the proportionality and relationship of IL-8, IL-6 and TNF-α with oral cancer. Analysis of saliva and serum both were carried out with significant changes noted (*p*-value < 0.05). Additionally, Figure 2 explains how the activation of IL-8, IL-6 and TNF-α takes place through activated macrophages. Figure 2 also illustrates the range of their signaling pathways in tumor microenvironments, which are activated by, for example, IL-8 via chemokine receptors CXCR1 and CXCR2, which activate signal transducers and activators of transcription-3 (*STAT3*) and β-catenin, which has been shown to promote cell proliferation and angiogenesis. IL-6 receptor leads to the activation of the Janus kinases (JAK) and signal transducers and activators of transcription (*STATs*), which then stimulate pathways involving mitogen-activated protein kinase (MAPK), which in turn supports cancer development. While tumor necrosis factor receptor type 1-associated DEATH domain protein in humans is encoded by the *TRADD* gene, which recruits TNF receptor-associated factor-2, a protein in humans is encoded by the *TRAF2* gene TRAF2 and signaling molecule RIP which activates and induces Nuclear Factor Kappa-light-chain-enhancer of activated B Cells (NF-κB) pathway, which gets involved in cell survival and proliferation and anti-apoptotic factors, which explains the major role in the development of oral cancer.

Since 1863, Rudolf Virchow had claimed cancer to be a progressive, untreated form of chronic inflammation. Currently, it is widely accepted that chronic inflammation leads to approximately a quarter of the total malignancies diagnosed [31]. This establishes the link between cytokines, which pro-inflammatory in nature, and cancer. Typically, the cytokine group would induce tissue repair and healing. However, in cancerous cells, they induced DNA damage, inhibition of DNA repair, inactivation of tumor suppressor genes functionality, vascular permeability, extravasation of fibrin, tissue remodeling, tumor cell migration, leukocyte infiltration, alteration of cell to cell adhesion molecules, decreased the immune response, and angiogenesis [20].

## 3. Salivary IL-8, IL-6 and TNF-α Role in Oral Cancer Diagnosis

In the past decade, saliva has emerged as a medium for disease analysis, including local and systemic conditions. Many investigators use saliva collected by simple drool technique. The cytokines were then analyzed quantitatively and qualitatively by enzyme-linked immunosorbent assay (ELISA) and polymerase chain reaction (PCR), respectively. Other tests utilized are Western blotting, migration assay, immuno-histochemical staining, spectrophotometer and neutrophil count, assessed either by Giemsa staining or culture. A comparison of the IL-8, IL-6 and TNF-α levels in serum and saliva have also been carried out, in which the results obtained showed equal values of IL-8 in both mediums. This highlights the use of saliva rather than serum. A comparative table showing the details of the research conducted, patients evaluated, results and p-values is listed below (Table 1). Khyani et al. [32] evaluated the salivary levels of IL-6 and IL-8 in patients diagnosed with histologically proven OSCC, potentially malignant lesions (PML), and a control group. The results revealed that in the OSCC group both biomarkers were found to be statistically elevated when compared with the control group. On the other hand, in the PML group, IL-8 was only found to be elevated [32]. However, it has been noted that the levels of IL-8 were found to be similar in both serum and salivary samples, whereas IL-6 was found to be higher in serum when compared to salivary samples of patients diagnosed with OSCC [33]. In another study conducted by Saheb et al., TNF-α, IL-8 and IL-6 levels were compared between patients diagnosed with OSCC, and age and sex matched controls. Only IL-6 levels were found to be statistically elevated. The other biomarkers, although raised compared to the control group, showed no statistically significant difference [34]. On the contrary, the levels of the same cytokines were evaluated in a study by Kaur et al. in salivary samples obtained from histologically proven patients of lichen planus, leukoplakia and oral submucous fibrosis (OSF), all three representing PML. The results obtained showed statistically significant, higher levels of IL-8 when compared to the control group. Also, the serum and the salivary samples, when compared, revealed a strong correlation amongst the groups [35]. In oral leukoplakia patients, salivary samples were studied, and the levels of salivary interleukin-6 and TNF-α were found to be significant as clinical diagnostic markers [36].

**Figure 3:**
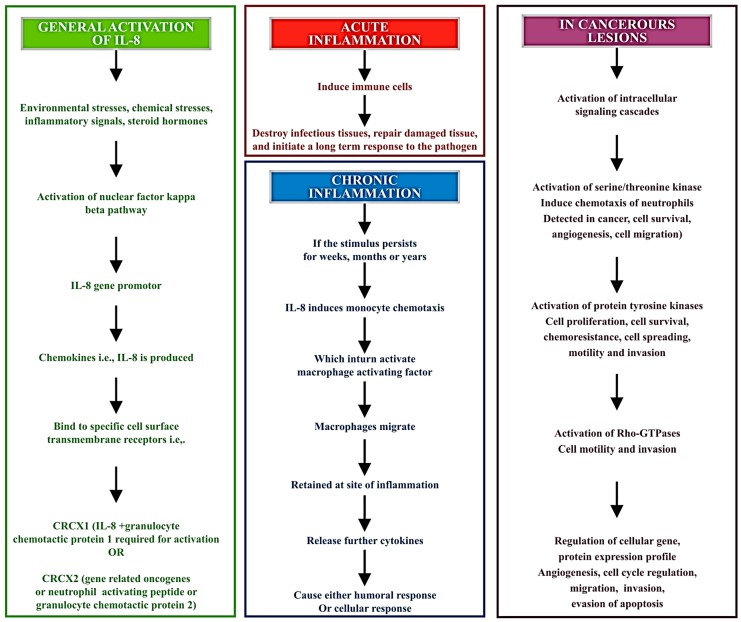
Pathways involved in activation and release of IL-8 and their effects at cellular level.

The levels obtained in samples from OSCC patients have been noted to be several times higher when compared to a control group, outweighing any other potential cause of the increase in levels. IL-8 levels may alter with lifestyle, geographical distribution, ethnic differences, genetic differences, and peculiar habits of individuals. Also, the levels may be found to be raised in mere gingivitis and periodontitis. Yet, none of these reasons can raise the levels to the extent seen in OSCC cases. Punyani et al. [12] discussed the pro-inflammatory and pro-angiogenic features of IL-8 through performing experiments with samples from 25 patients of OSCC, and hence found increased levels of IL-8 that confirmed the role of IL-8 in tumor angiogenesis and progression [12]. Furthermore, another reported work identified salivary and serum levels of IL-8, IL-6 and TNF-α in patients of oral leukoplakia, oral lichen planus, oral submucous fibrosis, and their results showed high concentrations of all cytokines, which again proved to be diagnostic markers for oral precancerous lesions [35]. A definitive alteration in the levels of IL-8 is noted from this; we can easily derive that IL-8 has a major potential to be used as a single salivary biomarker. This can open a new horizon for treatment plans of targeted leukotriene therapy [21,22]. The pathogenesis of the biomarker is elaborated upon in Figure 3, below.

## 4. Conclusions

The detection of cytokines in the saliva of cancerous patients has led to the conclusion that chemokines are held responsible, amongst the many other pro-inflammatory cytokines, for inducing oral cancer. Using saliva as a liquid media for evaluation has its unique advantages, including ease of handling and performing, compliance of patients and, in short, being a cost-effective tool for diagnosing, screening and assessment of OSCC treatment. These biomarkers can be utilized as a major asset for early detection as their role in tumorigenesis is exceedingly evident. Unfortunately, the results from various studies are still incomplete; numerous studies need to be conducted to form an accurate statement.

## Figures and Tables

**Table 1:** Description of reported studies on salivary IL-8 as an oral cancer biomarker.

Refrences	Patients	Sample Type	Sample Collection Time	Type of the Study	Analysing Tools Name	Outcome of the Study	*p* Value
[30]	Patients with newly diagnosed T1 or T2 oral cavity or oropharyngeal histologically confirmed squamous cell carcinoma were recruited for the study.	WMUS	Not mentioned	ECS	PCR, ELISA	Findings indicate that IL-8 in saliva and IL-6 in serum hold promise as biomarkers for OSCC. A saliva-based test could be a cost-effective adjunctive tool in the diagnosis and follow-up of patients with OSCC.	Interleukin-8 was detected at higher concentrations in saliva (*p* < 01) and IL-6 was detected at higher concentrations in serum of patients with OSCC (*p* < 0.01).
[35]	54 oral lichen planus, 50 oral leukoplakia, 51 oral submucous fibrosis, and 50 healthy controls.	WMUS	9:00 and 10:00 a.m.	ECS	ELISA	Salivary and serum IL-8, IL-6, and TNF-α levels might act as diagnostic markers for the detection of oral precancer.	The levels of serum and salivary TNF-α, IL-6, and IL-8 were statistically significantly increased in oral leukoplakia, sub-mucous fibrosis, and lichen planus in contrast to normal healthy subjects (*p* < 0.05). Serum and salivary correlation analysis revealed strong and highly significant correlations for TNF-α, IL-6, and IL-8 in all groups (r = 0.72–0.82, *p* < 0.05).
[34]	Nine patients with oral squamous cell carcinomas and healthy controls.	WMUS	9:00 and 11:00 a.m.	ECS	ELISA	Results shows that more studies are needed to accept the utility of these cytokines in predicting or diagnosis of oral squamous cell carcinoma or evaluation of treatment.	The concentration of salivary tumor necrosis factor α, interleukin-1α and 8 in case group was higher than control group, but it was not statistically significant (*p* > 0.05).
[12]	Oral pre-cancer and oral squamous cell carcinoma (OSCC) patients were compared with healthy controls.	WMUS	Not mentioned	ECS	ELISA	Results suggested that salivary IL-8 can be utilized as a potential biomarker for OSCC. Salivary IL- 8 was found to be non-conclusive for oral pre-malignancy in this preliminary study.	The levels of salivary IL-8 were found to be significantly elevated in patients with OSCC as compared to the pre-cancer group (*p* < 0.0001) and healthy controls (*p* < 0.0001). However, the difference in salivary IL-8 concentrations among the pre-cancer group and controls was not statistically significant.
[33]	50 patients in total, with 30 diagnosed with OSCC and 20 healthy controls.	Serum and salivary analysis	10 a.m.–1 p.m.	ECS	ELISA	Salivary IL-1α and Granulocyte macrophage colony stimulating factor GM-CSF was useful in the diagnosis of OSCC patients. Serum IL-6 was more useful in the diagnosis of OSCC patients than salivary IL-6. Serum and salivary IL-8 were very useful in the diagnosis of OSCC patients and for identifying between OSCC patients and the control group.	Serum IL-6 and IL-8 levels were detected at higher concentrations in patients with OSCC than in the control group (*p* < 0.001).
[37]	105 cases total; A = PMD, B = OSSC, C = Healthy controls; 35 in each group	Salivary analysis	Not mentioned	ECS	ELISA	The values were found to be consistently raised in groups A and B.	Statistically significant association between the groups with regards to IL-8 levels.
[32]	105 cases total; A = PMD, B = OSSC, C = Healthy controls; 35 in each group	Salivary analysis	Not mentioned	ECS	ELISA	The values were found to be consistently raised in groups A and B.	Statistically significant association between the groups with regards to IL-8 levels.

WMUS = whole mouth unstimulated saliva, ECS = experimental cross section, ELISA = enzyme linked immunosorbent assay, PCR = polymerase chain reaction; OSCC = oral squamous cell carcinoma.
